# Supplementary material for: Seq2Neo: A Comprehensive Pipeline for Cancer Neoantigen Immunogenicity Prediction
Source: Int J Mol Sci. 2022 Oct 1;23(19):11624. doi: 10.3390/ijms231911624 (PMC9569519; doi:10.3390/ijms231911624)
Supplement: Supplementary file 1 [file ijms-23-11624-s001.zip › ijms-1897508-supplementary.pdf]

Seq2Neo: a comprehensive pipeline for cancer neoantigen immunogenicity prediction

Kaixuan Diao[1-3,†], Jing Chen[1-3,†], Tao Wu[1], Xuan Wang[1], Guangshuai Wang[1], Xiaoqin Sun[1], Xiangyu Zhao[1], Chenxu Wu[1], Jinyu Wang[1], Huizi Yao[1], Casimiro Gerarduzzi[4] and Xue-Song Liu[1,*]

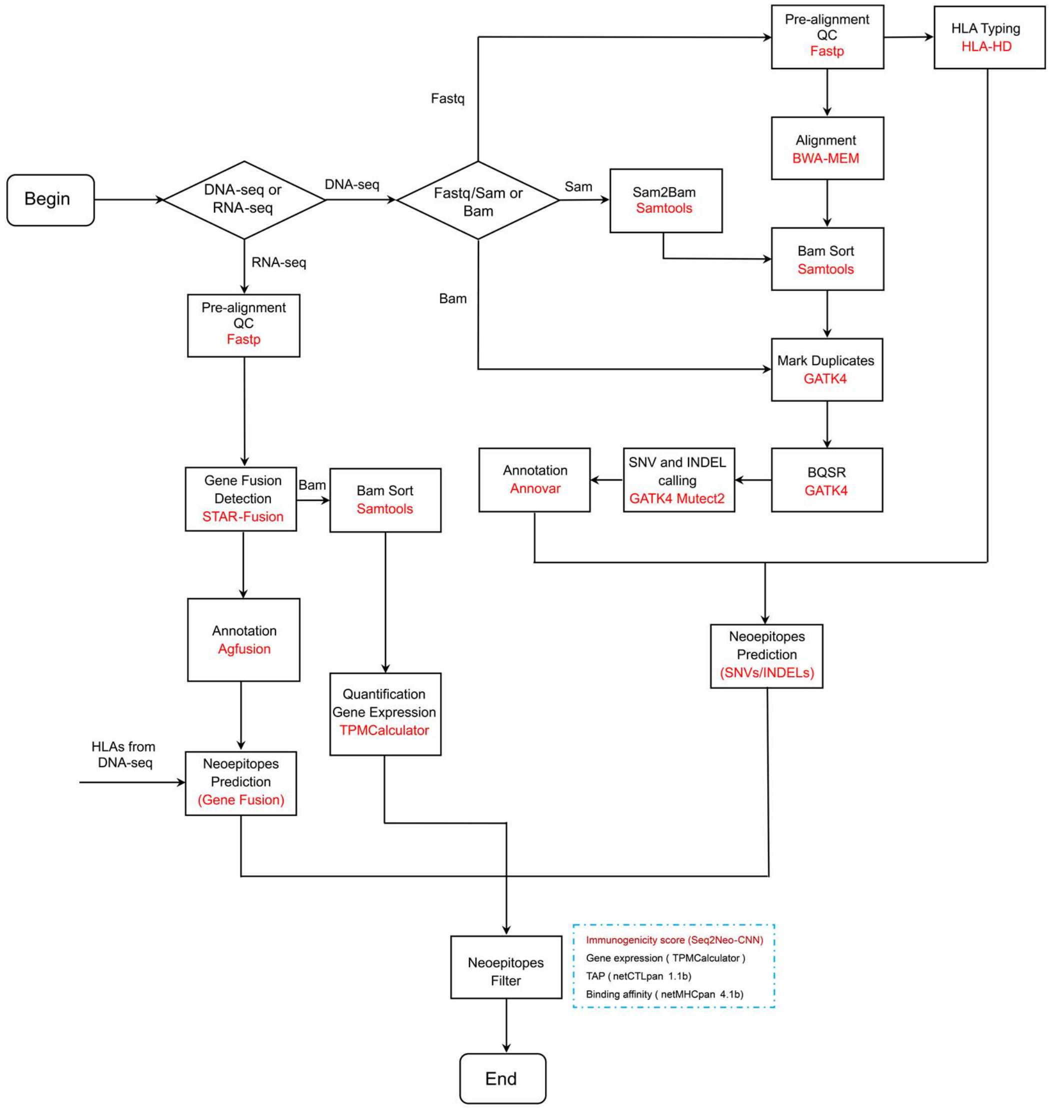

Figure S1. The detailed workflow of Seq2Neo. The detailed schematization of the Seq2Neo pipeline, each process and corresponding software are listed.

Algorithm	Top - 20	Top - 50
ExtraTrees	1	2
Random Forest	1	2
Logistic Regression	1	3
SVM	3	5
XGBoost	1	4
Seq2Neo-CNN	4	6

Figure S2. Comparisons of Seq2Neo-CNN and other machine learning algorithms. Comparisons of immunogenicity predictions on six machine learning classifiers, with the number of true-positive predictions overlapping each algorithm's top 20 or 50 predictions in the independent TESLA dataset.

A

Reference genome	Patient ID	#SNVs	#INDELs	#Fusions
GRCh38 Ensemble release 105	3998	213	14	2
	3942	119	10	4
	3995	59	8	0
	4032	107	12	3
	4166	171	3	1

B

Tumor Type	Patient ID	Validated Mutation Sites
Melanoma	3998	MAGEA6-E168K<br>PDS5A-H1007Y<br>MED13-P1691S
Rectal	3942	GPD2-E426K<br>NUP98-A359D<br>KARS-D328H
Colon	3995	RNF213-N1702S<br>TUBGCP2-P265L<br>KRAS-G12D
	4032	API5-R243Q<br>PHLPP1-G566E<br>RNF10-E572K
	4166	NPLOC4-I473V<br>SUN1-A127T

C

Patient ID	HLA	Peptide	TAP	IC50	Immuno	TPM	Gene	Change	Rank	Percentage	pVACseq Percentage	TSNAD 2.0 Percentage	NeoPredPipe Percentage
3998	HLA-A30:02	SVQIISCQY	3.131	55.08	0.7258	8.7023	MED13	P1691S	42	0.1842	0.4706	NA	0.2119
3998	HLA-A30:02	KVDPIGHVY	3.030	55.63	0.8232	4.4051	MAGEA6	E168K	43	0.1886	0.4853	0.4701	0.2161
3998	HLA-A01:01	MKVDPIGHVY	3.147	74.73	0.6652	4.4051	MAGEA6	E168K	57	0.2500	NA	NA	0.2754
3998	HLA-A30:02	VSVQIISCQY	3.115	76.23	0.8130	8.7023	MED13	P1691S	58	0.2544	NA	NA	0.2786
3942	HLA-C08:02	FGDVGSTLF	1.967	15.27	0.7359	0.5518	NUP98	A359D	7	0.0722	0.0938	NA	0.0596
3942	HLA-C08:02	FGDVGSTL	0.387	68.69	0.7433	0.5518	NUP98	A359D	23	0.2371	NA	NA	0.2553
3942	HLA-C16:01	ISKSGLITI	0.457	178.82	0.8189	0.4029	GPD2	E426K	57	0.5876	0.5625	0.5423	0.5574
3942	HLA-C08:02	VVDISKSGL	0.956	248.77	0.6974	0.4029	GPD2	E426K	66	0.6804	NA	NA	0.7021
3995	HLA-B18:01	MEHFYLSFY	2.998	8.54	0.5300	0.3604	RNF213	N1702S	1	0.0263	NA	NA	NA
3995	HLA-A18:01	MEHFYLSF	2.608	10.31	0.5479	0.3604	RNF213	N1702S	2	0.0526	NA	NA	0.0145
3995	HLA-A30:02	RILLVAASY	3.292	45.59	0.6521	3.2718	TUBGCP2	P256L	11	0.2895	0.3000	0.1834	0.1657
3995	HLA-A30:02	HRILLVAASY	3.156	199.70	0.6445	3.2718	TUBGCP2	P256L	25	0.6579	NA	0.7210	0.5407
4032	HLA-A02:13	LLQCTQQAV	0.438	26.87	0.5670	10.5973	API5	R243Q	7	0.0432	NA	0.0254	0.0994
4032	HLA-A02:13	RLLQCTQQAV	0.457	42.37	0.5380	10.5973	API5	R243Q	11	0.0679	NA	0.0522	0.1551
4032	HLA-A03:01	YLSHLPLTCK	0.310	44.20	0.6455	10.3660	RNF10	E577K	12	0.0741	NA	NA	0.1627
4032	HLA-A02:13	ILCETCLIV	0.413	128.13	0.6991	6.5426	PHLPP1	G566E	65	0.4012	0.7059	0.0501	0.4383
4032	HLA-B15:53	RQVILCETCL	1.484	267.47	0.7901	6.5426	PHLPP1	G566E	107	0.6605	NA	0.1963	NA
4032	HLA-A03:01	LSHLPLTCK	0.664	317.52	0.6905	10.3660	RNF10	E572K	121	0.7469	NA	NA	0.7846
4166	HLA-A02:01	SISQNPFPV	0.488	52.48	0.6424	6.0091	NPLOC4	I473V	53	0.2087	NA	0.2182	0.2624
4166	HLA-B07:02	FPVENRDVL	0.488	53.43	0.6714	6.0091	NPLOC4	I473V	55	0.2165	NA	NA	0.2667

Figure S3. Application of Seq2Neo in cancer patients. (A) Counts of different types of mutations in the five cancer samples calculated from Seq2Neo. (B) Experimentally verified neoantigenic sites of the five cancer samples. (C) Predicted neoantigens from the verified neoantigenic sites in the five cancer samples. The last three columns indicate the rank of neoantigens predicted by pVACseq, TSNAD 2.0 and NeoPredPipe, respectively. The lower rank of experimentally verified neoantigenic sites in Seq2Neo compared with other tools suggests the robustness of Seq2Neo in neoantigen prediction.
